# Nutrient Composition of Different Hazelnut Cultivars Grown in Germany

**DOI:** 10.3390/foods9111596

**Published:** 2020-11-03

**Authors:** Anke Katharina Müller, Ute Helms, Carsten Rohrer, Monika Möhler, Frank Hellwig, Michael Glei, Tanja Schwerdtle, Stefan Lorkowski, Christine Dawczynski

**Affiliations:** 1 Department of Nutritional Biochemistry and Physiology, Institute of Nutritional Sciences, Friedrich Schiller University Jena, Dornburger Str. 25, 07743 Jena, Germany; anke.katharina.mueller@uni-jena.de (A.K.M.); carsten.rohrer@uni-jena.de (C.R.); stefan.lorkowski@uni-jena.de (S.L.); 2 Competence Cluster for Nutrition and Cardiovascular Health (nutriCARD) Halle-Jena-Leipzig, Dornburger Str. 25, 07743 Jena, Germany; ute.helms@uni-jena.de (U.H.); michael.glei@uni-jena.de (M.G.); 3 Junior Research Group Nutritional Concepts, Institute of Nutritional Sciences, Friedrich Schiller University Jena, Dornburger Str. 29, 07743 Jena, Germany; 4 Department of Fruit Growing, Education & Research Institute of Horticulture, Leipziger Straße 75a, 99085 Erfurt, Germay; mon.moehler@lvg-erfurt.de; 5 Institute for Systematic Botany, Friedrich Schiller University Jena, Philosophenweg 16, 07743 Jena, Germany; frank.hellwig@uni-jena.de; 6 Department of Nutritional Toxicology, Institute of Nutritional Sciences, Friedrich Schiller University Jena, Dornburger Str. 24, 07743 Jena, Germany; 7 Department of Food Chemistry, Institute of Nutritional Science, University of Potsdam, Arthur-Scheunert-Allee 114–116, 14558 Nuthetal, Germany; taschwer@uni-potsdam.de; 8 Competence Cluster Nutrition Research (NutriAct), Berlin-Potsdam, Arthur-Scheunert-Allee 114–116, 14558 Nuthetal, Germany

**Keywords:** *Corylus avellana* L., nutrient composition, hazelnut cultivars, minerals, tocopherols

## Abstract

Hazelnuts are rarely cultivated in Germany, although they are a valuable source for macro- and micronutrients and can thus contribute to a healthy diet. Near the present, 15 varieties were cultivated in Thuringia, Germany, as a pilot study for further research. The aim of our study was to evaluate the micro- and macronutrient composition of representative, randomly mixed samples of the 15 different hazelnut cultivars. Protein, fat, and fiber contents were determined using established methods. Fatty acids, tocopherols, minerals, trace elements, and ultra-trace elements were analyzed using gas chromatography, high-performance liquid chromatography, and inductively coupled plasma triple quadrupole mass-spectrometry, respectively. We found that the different hazelnut varieties contained valuable amounts of fat, protein, dietary fiber, minerals, trace elements, and α-tocopherol, however, in different quantities. The variations in nutrient composition were independent of growth conditions, which were identical for all hazelnut varieties. Therefore, each hazelnut cultivar has its specific nutrient profile.

## 1. Introduction

Hazelnuts (*Corylus avellana* L., cobnut, including *Corylus maxima* Mill., Lambert Filbert) are popular tree nuts used in the human diet, which are mainly produced in Turkey followed by Italy and Spain [1]. Commercial hazelnut cultivation in Germany is rare, although hazelnuts are getting more popular and are often an essential ingredient in food production, e.g., in confectionery industry [2]. There are several reasons for the rare production, such as uncertain profitability forecasts for this region, long yield times, and missing organizational structures or practical experience. However, climatic conditions are changing.

Nut consumption is regularly recommended worldwide due to the beneficial health effects of nuts, and nuts are an essential part of the Mediterranean diet [3]. The prevention of cardiovascular diseases by consuming tree nuts, in particular hazelnuts, were investigated widely and indicate positive effects most notably based on improved blood lipid profiles [4]. Hazelnuts contain appreciable amounts of macronutrients, such as fat, protein, and fiber, but also micronutrients, such as minerals and vitamins. Hazelnuts have a high content of monounsaturated fatty acids (MUFA) and contain relatively small amounts of saturated fatty acids (SFA). Hazelnuts are characterized by a particularly high concentration of oleic acid with contents up to 70% of all fatty acids (FA), followed by linolic acid and palmitic acid. Schlörmann et al. measured fiber levels of 8.7% for hazelnuts, which indicate that tree nuts are a good natural source of dietary fiber [5]. This high fiber content is discussed to be partly responsible for the inverse association of nut consumption and gaining weight [6]. Other important components are vitamin E, with α-tocopherol (αTOH) as the most abundant form with up to 40.6 mg/100 g [7], and minerals such as magnesium, calcium, potassium, copper, and iron [5,8]. However, not all nuts have the same contents of these ingredients, so the recommendation is to consume a mixture with a variety of different nuts and intake amounts up to 42.5 g per day [9].

The aim of this pilot study was therefore to evaluate 15 European cultivars grown in Thuringia in Germany regarding their nutritional value and to identify the most useful cultivar for large-scale hazelnut plantings, because we expected variety-dependent nutrient differences. Hence, the protein, fiber, and fat contents of these different hazelnut cultivars were determined. In addition, the FA distribution, ash and mineral content, and data on αTOH amounts are presented.

## 2. Materials and Methods

### 2.1. Samples

**Figure 1:**
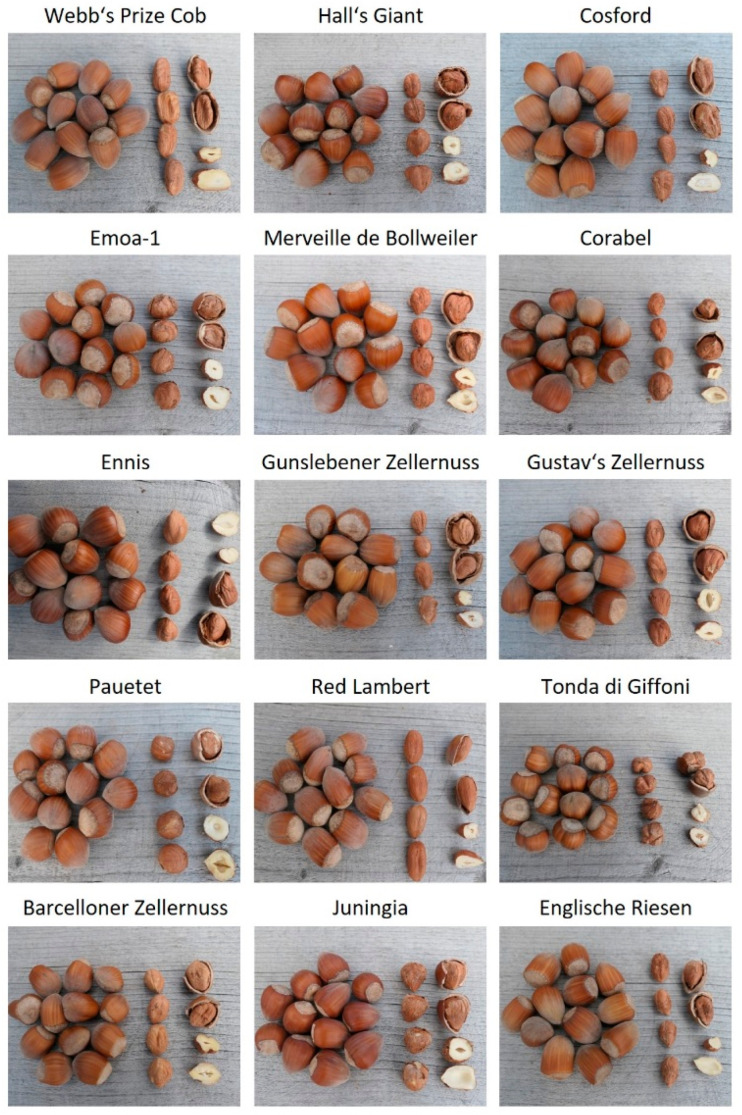
Representative exemplary pictures of the hazelnut cultivars studied.

In this study, varieties from different areas of origin were tested for cultivation. The varieties came from Germany, England, Spain, or Italy. The investigated 15 hazelnut (*Corylus avellana* L.) cultivars (Tonda di Giffoni, Juningia, Ennis, Cosford, Rote Lambert (Red Lambert), Englische Riesen, Webbs Preisnuss (Webb’s Prize Cob), Gustav Zeller (Gustav’s Zellernuss), Pauetet, Corabel, Hallesche Riesen (Hall’s Giant), Wunder aus Bollweiler (Merveille de Bollwiller), Gunslebert (Gunslebener Zellernuss), Emoa-1, Eckige Barceloner (Barcelloner Zellernuss)) were grown in experimental orchards in the region of Thuringia in Germany in 2005 (Figure 1). There is an ongoing debate about the status of *Corylus maxima* Mill. Lambert Filbert, which is either classified as an individual species or is considered as *Corylus avellana*. Since there is no concluding evidence which would favor the separation as an individual species and because of reported hybridization between both taxa, we do not discriminate between both species here. For a recent critical evaluation on *Corylus* taxonomy, we refer to Holstein et al. [10]. For synonymous names of cultivars, the reader is referred to Mehlenbacher [11], NCGR-Corvallis *Corylus* catalogue [12], and information provided by the Food and Agriculture Organization of the United Nations (FAO) [13]. The technical basis for the successful cultivation of hazelnuts in Germany was laid by many years of attempts by the Bavarian Department of Food, Agriculture, and Forestry in Fürth, Germany. To allow for reliable evaluations of the different hazelnut varieties, agronomical conditions were identical for the different cultivars: five trees of each cultivar were grown by the Department of Fruit Growing, Education, and Research Institute of Horticulture (Erfurt, Germany) for 12 years on a single testing ground in Thuringia, Germany. All cultivars were grown on their own roots except for the cultivar Ennis, which was grafted on *Corylus colurna* L. All cultivars were grown under identical treatment with drip irrigation and fertigation. Cultivation took place on comparable soil covered with loess. The mean planting distance between the five trees of a cultivar was about 4.5 m between the rows and about 2.5 m between the trees. Nuts were harvested in the same season of the same year but depending on differences in maturing times of each cultivar as assessed by independent qualified experts. The crop was carried out in 2016 and varied from 0.9 kg (average amount of Ennis) to 7.6 kg (average amount of Barcelloner Zellernuss) per tree (data not shown). Selected data and observations on growth and yield of the hazelnut varieties evaluated here are presented in Supplemental Table S1. After harvesting, nuts were dried at 30 to 35 °C in the dark for at least 7 days and were stored in their shell until use. For all analyses, representative samples of 100 randomly selected and freshly grounded nuts of a cultivar were used with skin.

### 2.2. Quantification of Main Constituents of Hazelnuts

All chemical analyses of the samples were done in accordance with the official methods of the Association of Official Agricultural Chemists (AOAC) [14]. Hazelnut fat content was examined using a Soxhlet extractor with petroleum ether and crude protein (Nx6.25) content was determined using a Kjeldahl apparatus. The total dietary fiber content of the different fat-free hazelnut varieties was measured according to the AOAC-certified protocol [15], using the Merck total fiber assay kit (Merck, Darmstadt, Germany). To determine the ash content, defined amounts of hazelnuts were dried and then completely incinerated in a muffle furnace at 525 °C.

### 2.3. Fatty Acid Composition

Fatty acid analysis of nuts was performed using gas chromatography (GC-17 V3; Shimadzu Corporation, Kyoto, Japan) equipped with a flame ionization detector and an autosampler (AOC-5000), as described [16]. Fatty acid concentrations were expressed as percentage of the total area of all FA methyl esters (% of total fatty acid methyl esters, FAME) using GC solution software version 2.3 (Shimadzu).

### 2.4. Tocopherol Determination

All high-performance liquid chromatography grade solvents and TOH standards were purchased from Merck, (Darmstadt, Germany) and LGC (Wesel, Germany), respectively. According to DIN EN 12822, HPLC (LC-20 AT; Shimadzu) was used to measure TOH concentrations of the grounded and homogenized nuts. After saponification with potassium hydroxide and extraction with n-hexane, TOH were separated on an Eurospher 100-5 Diol Vertex Plus Column 250 × 4 mm (Knauer, Berlin, Germany) with a mixture of n-hexane/methyl t-butylether (98/2 v/m) as mobile phase at a flow rate of 1.5 mL/min. Isomers of TOH were determined using a fluorescence detector (λex 295 nm, λem 330 nm; RF-10A XL; Shimadzu) and quantified using external standard calibration curves. 

### 2.5. Quantification of Minerals, Trace and Ultra-Trace Elements

Hazelnut samples were digested with nitric acid in a closed microwave digestion system (Mars 6, CEM, Kamp-Lintfort, Germany) and multi-element quantification was carried out with an ICP-QQQ-MS 8800 mass spectrometer (Agilent, Waldbronn, Germany) [17]. Calcium, magnesium, manganese, iron, copper, zinc, and cadmium were measured on mass, and arsenic and molybdenum were analyzed in the mass-shift mode using oxygen as a reaction gas to eliminate interferences. Rhodium (1 µg/L) was used as internal standard and helium (3 mL/min) as collision gas. For selenium isotope dilution, the analysis was applied as described [18]. The nebulizer gas flow and parameters of lenses, Q1, collision cell, and Q2 were tuned daily for maximum sensitivity (oxide ratio <1.0% (140Ce16O+/140Ce+), double charged ratio <1.5% (140Ce++/140Ce+), background counts <0.1 cps). For quality assurance, the measurement blanks and recalibration check points were determined periodically every 20 samples. For the verification of the applied method, the certified fish muscle reference material ERM-BB422 (Joint Research Centre, European Commission, Geel, Belgium) was successfully analyzed.

### 2.6. Statistics

Replicates were measured as indicated in Table 1, Table 2, Table 3 and Table 4. The results were expressed as means with standard deviation (SD) or indicated otherwise in Table 1, Table 2, Table 3 and Table 4.

## 3. Results and Discussion

The aim of the present study was to comprehensively evaluate the nutrient profile of 15 hazelnut varieties cultivated in Germany by analyzing their protein, ash, fiber, and fat content. In addition, the FA distribution was measured and data on TOH, minerals, and trace elements were collected. The present data are in accordance with previously published data of nutrient profiles in hazelnuts. Though, most of these investigations were carried out in hazelnuts from Turkey. Local variances can be explained by differences in soil composition and weather conditions. For the nuts analyzed in the present study, agricultural conditions did not differ as they were grown on the same ground. Thus, the observed alterations in the nutrient profiles of the hazelnuts studied here likely depend on the cultivar. The data presented here indicate also a high nutritional value of the hazelnuts grown in Germany. Thus, harvesting hazelnuts in Germany, even on commercial scale, could be an interesting option for improving nutrient supply.

### 3.1. Fat, Crude Protein, Dietary Fiber, Moisture and Ash

Results for the nutritional properties of 15 hazelnut cultivars grown in Germany are shown in Table 1. Fat is the predominant component and the total fat content varied between the 15 cultivars. While the cultivar Red Lambert contained 64.8 g/100 g fat in relation to the fresh weight, the Corabel variety had a fat content of 47.9 g/100 g. This range is comparable with data published previously. Savage and McNeil compared six varieties grown in New Zealand and described a fat content of 54.6 to 63.2% [19]. Later reports on varietal differences in the fat content of hazelnuts in Turkey and other regions revealed similar results [2,20,21,22,23]. Taş and Gökmen reported comparable fat contents in a range of 58.1 to 68.9% for hazelnuts harvested in Turkey in 2014 [24], while another group found somewhat lower total fat contents of 53.4 to 63.5% in six hazelnut cultivars grown in Iran in 2010 [25].

Variations were also found for other macronutrients. The crude protein content was the highest in the Corabel variety (22.1 g/100 g), whereas the Cosford variety contained the lowest (10.2 g/100 g). These results are in line with that obtained in other studies, where protein contents of 12 to 22% have been found [2,19,21,23,26]. Amaral et al. investigated 19 cultivars grown in Portugal and reported lower protein contents ranging from 9.3 to 12.7% [20].

Compared to published values, we found higher contents of dietary fiber. The Webb’s Prize Cob variety had an outstanding content of 22.2 g/100 g, whereas the Barcelloner Zellernuss variety contained only 13.4 g/100 g. Other studies reported values in the range of 9.5 to 13.2% [19,27,28]. With an average of 16.6% for the hazelnuts studied here, and especially for the varieties Webb’s Prize Cob, Hall’s Giant (19.7 g/100 g), and Merveille de Bollweiler (19.5 g/100 g), we found remarkably higher dietary fiber contents.

The content of ash, which allows an estimation about the mineral content, was in the range of 1.9 g/100 g (Red Lambert) to 3.2 g/100 g (Gunslebener Zellernuss). Previous reports have shown similar results [2,19]. Locatelli et al. reported slightly lower ash contents in a range of 1.30 to 2.75% [23].

### 3.2. Fatty Acid Composition

The tested varieties showed both differences as well as similarities regarding their FA distribution (Table 2). Palmitic acid (C16:0) accounted for only around 5% in all studied hazelnut varieties. Furthermore, proportions of palmitoleic acid (C16:1c9; data not shown), stearic acid (C18:0), and α-linolenic acid (C18:3c9,c12,c15) did not exceed 2.1%. However, there were noticeable differences in the content of the major FA. Oleic acid (C18:1c9) content varied in the range of 65.1 to 81.7%. The variety Tonda di Giffoni had the highest content (81.7%), while the variety Corabel showed the lowest value (65.1%). Next, linoleic acid (C18:3c9,c12) content differed from 10.3% in the Tonda di Giffoni variety to 26.8% in Corabel. The differences in these FA account for a cultivar-specific pattern of MUFA (66.9 to 83.0%) and polyunsaturated FA (PUFA; 10.4 to 27.0%). The total SFA content did not exceed 7.2%. The total n-3 (omega-3) PUFA content was very low for all hazelnuts (<0.2%). The total n-6 (omega-6) PUFA value varied from 10.3 (Tonda di Giffoni) to 26.8% (Corabel), depending on the cultivar.

The FA distribution of the analyzed hazelnuts were in good agreement with data previously reported [23,25]. Specific characteristics are the slightly lower levels of stearic acid in our varieties, with the highest value of 2.1% for Pauetet, while Locatelli et al. reported contents up to 4.9% [23]. In addition, five varieties (Cosford, Englische Riesen, Webb’s Prize Cob, Corabel, and Gunslebener Zellernuss) have oleic acid contents of less than 70%, which are low compared to literature data. This results in an inversely related high content of linoleic acid in these varieties of >20%, which exceeds the values reported in previous publications [20,21,22,23,24,29]. In previous studies, mostly Turkish varieties or nuts cultivated in southern regions were examined. However, an earlier report on the FA distribution of hazelnut cultivars grown in Iran revealed values comparable to ours [25]. In this study, oleic acid content varied from 64.2 to 81.3% and linoleic acid from 10.0 to 21.1%. Bacchetta et al. reported significant differences between two crop years regarding the FA content of 75 hazelnut cultivars from different countries [30]. This indicates that multiple determinants can influence the nutrient composition and especially the proportions of FA.

### 3.3. Tocopherols

Table 3 shows that αTOH is the major form of vitamin E in hazelnuts. Only traces of the vitamers β-, γ-, and δTOH were detected with contents of less than 2 mg/100 g. The variety Juningia contained the highest concentration of αTOH (28.9 mg/100 g), while only 9.9 mg/100 g were found in the variety Hall’s Giant. Tocopherol levels found here are in accordance with data from the literature. With a mean of 17.5 mg/100 g, the results herein are comparable to data on hazelnuts from Turkey and Portugal [27,31]. Taş and Gökmen reported the differences between two crop years and described notable decreases in the concentration of TOH for the second year for 14 varieties grown in Turkey [24]. However, another group measured TOH contents in hazelnuts grown in Poland and reported 73.90 mg/100 g αTOH for unroasted hazelnuts [32]. A study comparing nutritional values of hazelnuts mainly collected in Turkey determined total TOH contents in the range from 25.8 to 69.8 mg/100 g in hazelnut kernel oils [33]. Environmental, local, and analytical conditions are possible explanations for these remarkable differences.

### 3.4. Micronutrients

Nuts are known as a good source for minerals, which can contribute to a healthy diet [8]. We measured the amount of magnesium, calcium, manganese, iron, copper, zinc, cadmium, selenium, and arsenic in the 15 hazelnut cultivars. There are considerable differences between the examined hazelnuts regarding mineral, trace, and ultra-trace element compositions (Table 4). Magnesium contents ranged from 148 ± 3 mg/100 g (Tonda di Giffoni) to 213 ± 5 mg/100 g (Merveille de Bollweiler). Köksal et al. determined 15 cultivars of hazelnuts grown in Turkey and measured similar magnesium levels of 144 to 224 mg/100 g [21]. Data from other studies confirm these results [27,34]. In addition, the nuts contained high amounts of calcium. Calcium contents ranged from 140 ± 2 (Ennis) to 247 ± 2 mg/100 g (Cosford) and fit well into the picture previously published for hazelnuts grown in Turkey [27,34]. The lowest manganese content was found in Red Lambert (0.682 ± 0.001 mg/100 g), and the highest was determined for Gunslebener Zellernuss (3.92 ± 0.02 mg/100 g). Özdemir et al. reported comparable results with a range from 1.4 to 2.6 mg/100 g for commercial Turkish hazelnuts [34]. Juningia had the lowest level of iron with a content of 2.88 ± 0.07 mg/100 g, while the highest amount was found in Gunslebener Zellernuss with 4.67 ± 0.03 mg/100 g. These values are comparable with data provided in the literature [2,21,34,35,36]. Contents of copper ranged from 0.764 ± 0.011 to 2.17 ± 0.03 mg/100 g with highest amounts in the variety Corabel and lowest in the variety Ennis and are similar to those reported by others [33]. Corabel contained also the highest levels of zinc (3.93 ± 0.06 mg/100 g), while the variety Juningia contained only 2.12 ± 0.003 mg/100 g; both values are comparable with those reported in the literature [21,36]. Until now, less is known about the content of the trace element molybdenum in hazelnuts. The recommended value for an adequate intake is 65 µg/day [37]. Özkutlu et al. measured between 0.09 to 0.31 mg/kg molybdenum in hazelnuts grown in Turkey [38]. Our hazelnut varieties can contribute to a sufficient intake with contents ranging from 0.109 ± 0.003 mg/100 g (Tonda die Giffoni) to 0.515 ± 0.008 mg/100 g (Merveille de Bollweiler). Levels of selenium are low in the examined samples with highest contents in Tonda di Giffoni and Pauetet with 5.10 ± 0.20 µg/100 g and 6.25 ± 0.51 µg/100 g, respectively. Hazelnuts grown in Turkey showed higher amounts of selenium [27,36], but it is important to note that the soil content for selenium is very low in Germany [39]. Cadmium was only detected at very low amounts in Gunslebener Zellernuss (0.91 ± 0.06 µg/100 g), all other samples were under the limit of quantitation (data not shown). Arsenic levels remained lower than 4 µg/100 g in all varieties, which complies with data for foods of terrestrial origin [40].

## 4. Conclusions

The data from this pilot study show differences in nutrient profiles depending on the varieties. The observed variations in nutrient composition were independent of growth conditions and climate, which were identical for all hazelnut varieties, as well as year-to-year changes. Just as growth conditions, technical processability, and resistance to pests, the nutrient content is only one of several parameters for assessing the suitability of a variety for commercial cultivation. Based on these parameters and nutrient profiles, Emoa-1, Corabel, Webb’s Prize Cob, Barcelloner Zellernuss, and Merveille de Bollweiler were selected for long-term studies which are ongoing. While Corabel was the variety with the highest protein content, Webb’s Prize Cob variety showed a high content of dietary fiber and Merveille de Bollweiler had the highest content of magnesium. On the other hand, Emoa-1 conceded reliably good harvests and aromatic nuts and Barcelloner Zellernuss distinguished due to a very high yield. Assessing nutrient profiles, growing behavior, and resistance to pests of these cultivars over time will allow for recommending suitable varieties, rootstocks, and cultivation systems for regional cultivation as well as changes in nutrient profiles from year to year.

## Figures and Tables

**Table 1:** Composition [g/100 g] of 15 hazelnut cultivars grown under identical conditions in Thuringia, Germany.

	Fat	Protein	Dietary Fiber	Ash	Moisture
Tonda di Giffoni	62.7 ± 1.0	16.5 ± 0.2	13.8 ± 0.4	2.1 ± 0.0	3.9 ± 0.0
Juningia	62.3 ± 0.7	11.7 ± 0.1	15.3 ± 0.1	1.9 ± 0.0	3.8 ± 0.0
Ennis	59.8 ± 0.6	12.4 ± 0.1	18.9 ± 1.1	2.2 ± 0.1	3.5 ± 0.0
Cosford	52.6 ± 0.8	10.2 ± 0.2	14.9 ± 0.4	2.8 ± 0.1	4.0 ± 0.4
Red Lambert	64.8 ± 0.8	10.6 ± 0.2	16.4 ± 0.8	1.9 ± 0.0	3.3 ± 0.0
Englische Riesen	51.9 ± 0.0	19.7 ± 0.2	14.9 ± 1.2	2.8 ± 0.1	4.5 ± 0.1
Webb’s Prize Cob	50.9 ± 0.1	15.9 ± 0.0	22.2 ± 0.8	2.7 ± 0.0	4.5 ± 0.1
Gustav’s Zellernuss	60.6 ± 1.3	14.3 ± 0.1	18.1 ± 1.8	2.6 ± 0.1	4.0 ± 0.1
Pauetet	57.7 ± 0.2	16.0 ± 0.0	14.4 ± 0.1	2.2 ± 0.0	3.9 ± 0.1
Corabel	47.9 ± 0.8	22.1 ± 0.1	14.7 ± 0.2	3.1 ± 0.0	4.4 ± 0.0
Hall’s Giant	54.1 ± 1.3	18.4 ± 0.1	19.7 ± 0.7	2.5 ± 0.0	4.0 ± 0.0
Merveille de Bollweiler	54.1 ± 1.4	14.2 ± 0.2	19.5 ± 0.2	2.7 ± 0.0	4.3 ± 0.0
Gunslebener Zellernuss	50.3 ± 0.3	17.4 ± 0.2	16.7 ± 0.8	3.2 ± 0.1	4.4 ± 0.0
Emoa-1	56.9 ± 0.3	15.1 ± 0.1	13.9 ± 0.6	2.6 ± 0.0	3.9 ± 0.0
Barcelloner Zellernuss	60.2 ± 0.2	16.1 ± 0.1	13.4 ± 0.5	2.2 ± 0.0	3.9 ± 0.1

Data refer to fresh weight; values are expressed as means ± SD (*n* = 2).

**Table 2:** Fatty acid composition (% of total FAME ^1^) of 15 hazelnut cultivars grown under identical conditions in Thuringia, Germany.

	C16:0	C18:0	C18:1 n-9	C18:2 n-6 (LA) ^2^	C-18:3 n-3 (ALA) ^3^	Σ SFA ^4^	Σ MUFA ^5^	Σ PUFA ^6^	Σ n-3	Σ n-6
Tonda di Giffoni	4.5	1.8	81.7	10.3	0.1	6.5	83.0	10.7	0.1	10.3
Juningia	4.7	1.8	81.0	10.5	0.1	6.8	82.6	10.7	0.1	10.5
Ennis	5.2	1.8	77.1	13.9	0.1	7.2	78.8	14.0	0.1	13.9
Cosford	4.7	1.2	68.1	24.1	0.2	6.0	69.7	24.3	0.2	24.1
Red Lambert	4.9	1.9	80.3	11.0	0.1	7.0	81.8	11.2	0.1	11.0
Englische Riesen	4.1	1.0	69.0	24.0	0.2	5.2	70.5	24.3	0.2	24.0
Webb’s Prize Cob	4.9	0.8	65.8	26.2	0.2	5.9	67.6	26.5	0.2	26.2
Gustav’s Zellernuss	4.4	1.7	76.4	15.6	0.1	6.3	77.9	15.8	0.1	15.6
Pauetet	4.7	2.1	80.8	10.6	0.1	7.0	82.2	10.8	0.1	10.6
Corabel	4.9	1.0	65.1	26.8	0.2	6.1	66.9	27.0	0.2	26.8
Hall’s Giant	4.3	1.7	75.2	16.9	0.1	6.3	76.6	17.1	0.1	16.9
Merveille de Bollweiler	4.4	1.4	77.0	15.3	0.1	6.0	78.6	15.5	0.1	15.3
Gunslebener Zellernuss	4.6	1.1	66.2	25.9	0.2	5.9	67.9	26.1	0.2	25.9
Emoa-1	4.6	1.8	72.1	19.5	0.1	6.6	73.6	19.8	0.1	19.5
Barcelloner Zellernuss	4.3	1.8	79.4	12.8	0.1	6.3	80.7	13.0	0.1	12.8

^1^ FAME, fatty acid methyl esters. ^2^ LA, linoleic acid. ^3^ ALA, α-linolenic acid. ^4^ SFA, saturated fatty acids. ^5^ MUFA, monounsaturated fatty acids. ^6^ PUFA, polyunsaturated fatty acids.

**Table 3:** Vitamin E content [mg/100 g] of 15 hazelnut cultivars grown under identical conditions in Thuringia, Germany.

	α-Tocopherol	β-Tocopherol	γ-Tocopherol	δ-Tocopherol
Tonda di Giffoni	13.5	<0.6	<1.0	<0.6
Juningia	28.9	0.80	<1.0	<0.6
Ennis	21.2	0.60	<1.0	<0.6
Cosford	20.7	<0.6	<1.0	<0.6
Red Lambert	24.8	<0.6	2.00	<0.6
Englische Riesen	16.6	0.60	<1.0	<0.6
Webb’s Prize Cob	16.3	<0.6	<1.0	<0.6
Gustav’s Zellernuss	13.3	<0.6	<1.0	<0.6
Pauetet	19.2	<0.6	<1.0	<0.6
Corabel	10.9	<0.6	<1.0	<0.6
Hall’s Giant	9.9	<0.6	<1.0	<0.6
Merveille de Bollweiler	11.8	<0.6	<1.0	<0.6
Gunslebener Zellernuss	18.6	<0.6	<1.0	<0.6
Emoa-1	15.6	<0.6	<1.0	<0.6
Barcelloner Zellernuss	16.1	<0.6	<1.0	<0.6

Data refer to fresh weight.

**Table 4:** Composition of minerals, trace, and ultra-trace elements of 15 hazelnut cultivars grown under identical conditions in Thuringia, Germany.

	Mg (mg/100 g)	Ca (mg/100 g)	Mn (mg/100 g)	Fe (mg/100 g)	Cu (mg/100 g)	Zn (mg/100 g)	Mo (mg/100 g)	Se (µg/100 g)	As (µg/100 g)
Tonda di Giffoni	148 ± 3	177 ± 4	2.78 ± 0.09	3.02 ± 0.04	1.14 ± 0.02	2.48 ± 0.03	0.109 ± 0.003	5.10 ± 0.20	1.57 ± 0.14
Juningia	155 ± 3	155 ± 1	1.22 ± 0.02	2.88 ± 0.07	0.948 ± 0.013	2.12 ± 0.003	0.310 ± 0.004	4.33 ± 0.28	0.95 ± 0.04
Ennis	162 ± 3	140 ± 2	1.17 ± 0.02	3.21 ± 0.02	0.764 ± 0.011	2.36 ± 0.05	0.414 ± 0.004	3.11 ± 0.11	2.01 ± 0.06
Cosford	178 ± 3	247 ± 2	2.94 ± 0.01	3.42 ± 0.03	1.26 ± 0.01	2.91 ± 0.03	0.256 ± 0.003	4.55 ± 0.45	2.53 ± 0.09
Red Lambert	151 ± 1	176 ± 1	0.682 ± 0.001	3.34 ± 0.03	1.21 ± 0.004	2.48 ± 0.01	0.231 ± 0.002	2.73 ± 0.21	2.57 ± 0.07
Englische Riesen	211 ± 1	241 ± 3	2.71 ± 0.02	3.09 ± 0.04	1.36 ± 0.01	2.87 ± 0.01	0.309 ± 0.002	4.49 ± 0.47	3.58 ± 0.04
Webb’s Prize Cob	173 ± 3	235 ± 3	1.40 ± 0.01	3.71 ± 0.05	1.06 ± 0.02	2.92 ± 0.01	0.331 ± 0.006	3.69 ± 0.23	3.47 ± 0.12
Gustav’s Zellernuss	206 ± 2	224 ± 1	2.10 ± 0.01	3.90 ± 0.004	1.84 ± 0.06	3.01 ± 0.01	0.479 ± 0.010	3.29 ± 0.27	3.81 ± 0.07
Pauetet	162 ± 2	175 ± 0.4	1.87 ± 0.02	3.73 ± 0.06	1.52 ± 0.02	3.02 ± 0.04	0.117 ± 0.001	6.25 ± 0.51	2.35 ± 0.06
Corabel	188 ± 5	212 ± 5	2.91 ± 0.05	4.26 ± 0.11	2.17 ± 0.03	3.93 ± 0.06	0.280 ± 0.005	4.79 ± 0.45	2.38 ± 0.07
Hall’s Giant	182 ± 1	211 ± 14	1.67 ± 0.10	3.67 ± 0.04	1.71 ± 0.05	3.40 ± 0.03	0.297 ± 0.005	2.94 ± 0.23	2.91 ± 0.07
Merveille de Bollweiler	213 ± 5	201 ± 6	1.77 ± 0.04	3.74 ± 0.12	1.85 ± 0.05	2.88 ± 0.05	0.515 ± 0.008	3.68 ± 0.05	2.13 ± 0.05
Gunslebener Zellernuss	209 ± 3	207 ± 1	3.92 ± 0.02	4.67 ± 0.03	1.79 ± 0.04	3.43 ± 0.03	0.351 ± 0.001	4.11 ± 0.56	2.69 ± 0.03
Emoa-1	180 ± 3	225 ± 1	1.94 ± 0.04	3.49 ± 0.05	0.972 ± 0.010	2.81 ± 0.02	0.279 ± 0.004	4.23 ± 0.11	1.99 ± 0.07
Barcelloner Zellernuss	171 ± 6	232 ± 11	2.33 ± 0.10	3.99 ± 0.29	0.779 ± 0.027	2.68 ± 0.11	0.204 ± 0.003	3.83 ± 0.26	2.61 ± 0.16

Data refer to fresh weight; values are expressed as means ± SD (*n* = 3).

## Supplementary Materials

The following are available online at https://www.mdpi.com/2304-8158/9/11/1596/s1, Table S1: Agronomic data of and observations for the 15 varieties cultivated in Germany.

## References

[1] FAOSTAT (2016). Value of Agricultural Production.

[2] Ozdemir F., Akinci I. (2004). Physical and nutritional properties of four major commercial Turkish hazelnut varieties. J. Food Eng..

[3] Ros E. (2015). Nuts and CVD. Br. J. Nutr..

[4] Perna S., Giacosa A., Bonitta G., Bologna C., Isu A., Guido D., Rondanelli M. (2016). Effects of Hazelnut Consumption on Blood Lipids and Body Weight: A Systematic Review and Bayesian Meta-Analysis. Nutrients.

[5] Schlormann W., Birringer M., Böhm V., Löber K., Jahreis G., Lorkowski S., Müller A., Schöne F., Glei M. (2015). Influence of roasting conditions on health-related compounds in different nuts. Food Chem..

[6] Jackson C.L., Hu F.B. (2014). Long-term associations of nut consumption with body weight and obesity. Am. J. Clin. Nutr..

[7] Stuetz W., Schlörmann W., Glei M. (2017). B-vitamins, carotenoids and α-/γ-tocopherol in raw and roasted nuts. Food Chem..

[8] Ros E. (2010). Health Benefits of Nut Consumption. Nutrients.

[9] Administration F.D., FDA (2003). Qualified Health Claims: Letter of Enforcement Discretion Nuts and Coronary Heart Disease (Docket No 02p-0505).

[10] Holstein N., El Tamer S., Weigend M. (2018). The nutty world of hazel names—a critical taxonomic checklist of the genus Corylus (Betulaceae). Eur. J. Taxon..

[11] Mehlenbacher S.A. (2008). Betulaceae Corylus.

[12] NCGR-Corvallis Ncgr-Corvallis Corylus Catalog. https://www.ars.usda.gov/ARSUserFiles/20721500/catalogs/corcore.html.

[13] Köksal A.I. (2000). Inventory of Hazelnut Research, Germplasm and References.

[14] AOAC (2012). Official Methods of Analysis of Aoac International.

[15] Lee S.C., Prosky L., De Vries J.W. (1992). Determination of Total, Soluble, and Insoluble Dietary Fiber in Foods—Enzymatic-Gravimetric Method, MES-TRIS Buffer: Collaborative Study. J. AOAC Int..

[16] Dawczynski C., Schubert R., Jahreis G. (2007). Amino acids, fatty acids, and dietary fibre in edible seaweed products. Food Chem..

[17] Meyer S., Markova M., Pohl G., Marschall T.A., Pivovarova O., Pfeiffer A.F., Schwerdtle T. (2018). Development, validation and application of an ICP-MS/MS method to quantify minerals and (ultra-)trace elements in human serum. J. Trace Elements Med. Biol..

[18] Marschall T.A., Kroepfl N., Jensen K.B., Bornhorst J., Meermann B., Kuehnelt D., Schwerdtle T. (2017). Tracing cytotoxic effects of small organic Se species in human liver cells back to total cellular Se and Se metabolites. Metallomics.

[19] Savage G., McNeil D.L. (1998). Chemical composition of hazelnuts (Corylus avellana L.) grown in New Zealand. Int. J. Food Sci. Nutr..

[20] Amaral J.S., Casal S., Citová I., Santos A., Seabra R.M., Oliveira B.P.P. (2005). Characterization of several hazelnut (Corylus avellana L.) cultivars based in chemical, fatty acid and sterol composition. Eur. Food Res. Technol..

[21] Köksal A.I., Artik N., Şimşek A., Güneş N. (2006). Nutrient composition of hazelnut (Corylus avellana L.) varieties cultivated in Turkey. Food Chem..

[22] Alasalvar C., Pelvan E., Topal B. (2010). Effects of roasting on oil and fatty acid composition of Turkish hazelnut varieties (Corylus avellanaL.). Int. J. Food Sci. Nutr..

[23] Locatelli M., Coïsson J.D., Travaglia F., Bordiga M., Arlorio M. (2015). Impact of Roasting on Identification of Hazelnut (Corylus avellana L.) Origin: A Chemometric Approach. J. Agric. Food Chem..

[24] Taş N.G., Gökmen V. (2015). Profiling triacylglycerols, fatty acids and tocopherols in hazelnut varieties grown in Turkey. J. Food Compos. Anal..

[25] Rezaei F., Bakhshi D., Fotouhi Ghazvini R., Javadi Majd D., Pourghayoumi M. (2014). Evaluation of fatty acid content and nutritional properties of selected native and imported hazelnut (corylus avellana l.) varieties grown in iran. J. Appl. Bot. Food Qual..

[26] Bonvehí J.S. (1995). A chemical study of the protein fractions of Tarragona hazelnuts (Corylus avellana). Eur. Food Res. Technol..

[27] Alasalvar C., Shahidi F., Liyanapathirana C.M., Ohshima T. (2003). Turkish Tombul Hazelnut (Corylus avellanaL.). 1. Compositional Characteristics. J. Agric. Food Chem..

[28] Dodevska M., Sobajic S., Djordjevic B. (2015). Fibre and polyphenols of selected fruits, nuts and green leafy vegetables used in Serbian diet. J. Serbian Chem. Soc..

[29] Ciemniewska-Żytkiewicz H., Verardo V., Pasini F., Bryś J., Koczoń P., Caboni M.F. (2015). Determination of lipid and phenolic fraction in two hazelnut (Corylus avellana L.) cultivars grown in Poland. Food Chem..

[30] Bacchetta L., Aramini M., Zini A., Di Giammatteo V., Spera D., Drogoudi P., Rovira M., Silva A.P., Solar A., Botta R. (2013). Fatty acids and alpha-tocopherol composition in hazelnut (Corylus avellana L.): A chemometric approach to emphasize the quality of European germplasm. Euphytica.

[31] Amaral J.S., Casal S., Alves M.R., Seabra R.M., Oliveira M.B.P.P. (2006). Tocopherol and Tocotrienol Content of Hazelnut Cultivars Grown in Portugal. J. Agric. Food Chem..

[32] Marzocchi S., Pasini F., Verardo V., Ciemniewska-Żytkiewicz H., Caboni M.F., Romani S. (2017). Effects of different roasting conditions on physical-chemical properties of Polish hazelnuts (Corylus avellana L. var. Kataloński). LWT.

[33] Matthäus B., Özcan M.M. (2012). The comparison of properties of the oil and kernels of various hazelnuts from Germany and Turkey. Eur. J. Lipid Sci. Technol..

[34] Özdemir M., Açkurt F., Kaplan M., Yıldız M., Loker M., Gürcan T., Biringen G., Okay A., Seyhan F.G. (2001). Evaluation of new Turkish hybrid hazelnut (Corylus avellana L.) varieties: Fatty acid composition, α-tocopherol content, mineral composition and stability. Food Chem..

[35] Açkurt F., Ozdemir M., Biringen G., Loker M. (1999). Effects of geographical origin and variety on vitamin and mineral composition of hazelnut (Corylus avellana L.) varieties cultivated in Turkey. Food Chem..

[36] Simsek A., Aykut O. (2007). Evaluation of the microelement profile of Turkish hazelnut (Corylus avellanaL.) varieties for human nutrition and health. Int. J. Food Sci. Nutr..

[37] EFSA Panel on Dietetic Products, Nutrition, and Allergies (NDA) (2013). Scientific Opinion on Dietary Reference Values for molybdenum. EFSA J..

[38] Özkutlu F., Ziya Doğru Y., Özenç N., Yazici G., Turan M., Akçay F. (2011). The importance of turkish hazelnut trace and heavy metal contents for human nutrition. J. Soil Sci. Environ. Manag..

[39] Utermann J., Fuchs M., Düwel O. (2008). Flächenrepräsentative Hintergrundwerte für arsen, Antimon, Beryllium, Molybdän, Kobalt, Selen, Thallium, Uran und Vanadium in Böden Deutschlands aus Länderübergreifender Sicht.

[40] Molin M., Ulven S.M., Meltzer H.M., Alexander J. (2015). Arsenic in the human food chain, biotransformation and toxicology—Review focusing on seafood arsenic. J. Trace Elements Med. Biol..

