# Supplementary material for: Nutrient Composition of Different Hazelnut Cultivars Grown in Germany
Source: Foods. 2020 Nov 3;9(11):1596. doi: 10.3390/foods9111596 (PMC7692035; doi:10.3390/foods9111596)
Supplement: Supplementary file 1 [file foods-09-01596-s001.pdf]

Supplemental material to

Nutrient Composition of Different Hazelnut Cultivars Grown in Germany

Anke Katharina Müller[1,2], Ute Helms[2,3], Carsten Rohrer[1,2], Monika Möhler[4], Frank Hellwig[5], Michael Glei[2,6], Tanja Schwerdtle[7,8], Stefan Lorkowski[1,2], Christine Dawczynski[1,2,3*]

1 Department of Nutritional Biochemistry and Physiology, Institute of Nutritional Sciences, Friedrich Schiller University Jena, Dornburger Str. 25, 07743 Jena, Germany; anke.katharina.mueller@uni-jena.de; carsten.rohrer@uni-jena.de; stefan.lorkowski@uni-jena.de; christine.dawczynski@uni-jena.de
2 Competence Cluster for Nutrition and Cardiovascular Health (nutriCARD), Halle-Jena-Leipzig, Dornburger Str. 25, 07743 Jena, Germany; ute.helms@uni-jena.de; michael.glei@uni-jena.de
3 Junior Research Group Nutritional Concepts, Institute of Nutritional Sciences, Friedrich Schiller University Jena, Dornburger Str. 29, 07743 Jena, Germany;
4 Department of Fruit Growing, Education & Research Institute of Horticulture, Leipziger Straße 75a, 99085 Erfurt, Germany; ~~mon.moehler@lvg-erfurt.de~~
5 Institute for Systematic Botany, Friedrich Schiller University Jena, Philosophenweg 16, 07743 Jena, Germany; frank.hellwig@uni-jena.de
6 Department of Nutritional Toxicology, Institute of Nutritional Sciences, Friedrich Schiller University Jena, Dornburger Str. 24, 07743 Jena, Germany;
7 Department of Food Chemistry, Institute of Nutritional Science, University of Potsdam, Arthur-Scheunert-Allee 114-116, 14558 Nuthetal, Germany; taschwer@uni-potsdam.de
8 Competence Cluster Nutrition Research (NutriAct), Berlin-Potsdam, Arthur-Scheunert-Allee 114 – 116, 14558 Nuthetal, Germany
* Correspondence: christine.dawczynski@uni-jena.de; Tel.: +49-3641-9-49656

Table S1 Agronomic data of and observations for the 15 varieties cultivated in Germany.

Variety	Vigorous growth	Stolon ungrafted	Yield	Observations
Emoa-1	Weak	Minimal	High, regular	Oblong to round fruits, reliably good harvests, aromatic
Webb's Prize Cob	Weak	Moderate	High, regular	Fruit does not fall out of the shell, aromatic
Gunslebener Zellernuss	Moderate	Minimal	High, regular	Oblong to round fruit, aromatic
Barcelloner Zellernuss	Moderate	Moderate	Very high	Fruit quality poor, little flavor, shapeless cores, inconsistent
Englische Riesen	Moderate	Minimal	Moderate	Very susceptible to gall mite infestation, not very aromatic
Red Lambert	Moderate	Very strong	Moderate	Fruit does not fall out of the shell, aromatic, strong stolon formation
Corabel	Strong	Moderate	Moderate	Inconsistent cores, often double, mostly 13 mm, aromatic
Cosford	Strong	Moderate	Weak to moderate	Oblong, aromatic
Tonda di Giffoni	Strong	Moderate	Variable	Yield strongly dependent on the weather, mostly 14,5 mm
Ennis	Strong	-	Moderate	Very large, prone to hazel drills, aromatic
Hall's Giant	Very strong	Moderate	High, regular	Uniform cores, aromatic
Merveille de Bollweiler	Very strong	Moderate	Moderate	Oblong to round
Gustav Zeller	Very strong	Minimal	High, regular	Not very aromatic, mostly 12,5 mm
Pauetet	Very strong	Moderate	Weak to moderate	Aromatic, small fruits
Juningia	Strong	Minimal	Weak to moderate	Yield so far not satisfactory, aromatic
